# Ceramide and phosphatidylcholine lipids‐based risk score predicts major cardiovascular outcomes in patients with heart failure

**DOI:** 10.1111/eci.14359

**Published:** 2024-11-23

**Authors:** Angelika Witoslawska, Jennifer M. T. A. Meessen, Mika Hilvo, Antti Jylhä, Faiez Zannad, Marianna Cerrato, Patrick Rossignol, Deborah Novelli, Kevin Duarte, Giovanni Targher, Roberto Latini, Nicolas Girerd, Reijo Laaksonen

**Affiliations:** ^1^ Finnish Cardiovascular Research Center University of Tampere Tampere Finland; ^2^ Department of Acute Brain and Cardiovascular Injury Institute for Pharmacological Research Mario Negri IRCCS Milan Italy; ^3^ Zora Biosciences Oy Espoo Finland; ^4^ VTT Technical Research Centre of Finland Ltd. Espoo Finland; ^5^ Centre d'Investigation Clinique Plurithémathique Pierre Drouin & Département de Cardiologie, Institut Lorrain du Coeur et des Vaisseaux CHU de Nancy France; ^6^ Department of Medicine University of Verona Verona Italy; ^7^ Metabolic Diseases Research Unit, IRCCS Sacro Cuore Don Calabria Hospital Negrar di Valpolicella, VR Italy

**Keywords:** cardiovascular event, ceramides, plasma biomarkers, risk score, sphingolipids

## Abstract

**Background:**

Ceramide and phosphatidylcholine lipids‐based risk score (CERT2) has shown a strong prognostic value in predicting cardiovascular (CV) events in patients with ischemic heart disease. This study aimed to investigate the prognostic value of CERT2 risk score in patients with heart failure (HF).

**Methods:**

The current study combines data for 4234 subjects from the COMMANDER‐HF trial and 1227 subjects from the GISSI‐HF trial, which enrolled patients with a history of HF. The CERT2 risk score was calculated for all the participants as previously described. The primary outcome was CV death, but all‐cause death and major adverse CV events (three‐point MACE) were analysed as well.

**Results:**

After adjustment for established CV risk factors and potential confounders, patients with the highest CERT2 risk category remained at almost three‐fold higher risk of CV death (COMMANDER‐HF: HR 2.80, 95% CI 2.18–3.60, GISSI‐HF: 2.84, 95% CI 1.70–4.74), all‐cause death (COMMANDER‐HF: HR 2.97, 95% CI 2.36–3.75, GISSI‐HF: 2.83, 95% CI 1.83–4.38) and MACE (COMMANDER‐HF: HR 2.73, 95% CI 2.20–3.38, GISSI‐HF: 2.67, 95% CI 1.67–4.26) compared to those with the lowest CERT2 risk category.

**Conclusions:**

The CERT2 risk score is strongly associated with an increased risk of CV death, all‐cause death and MACE in patients with HF.

## Graphical abstract

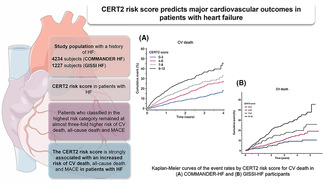

## 1 INTRODUCTION

Heart failure (HF) is a clinical syndrome resulting from a cardiac disorder that can be associated with many cardiovascular (CV) diseases, such as coronary artery disease (CAD).^1^ HF is becoming a major health crisis with over 60 million people affected globally.^2^ With an increasing global prevalence of HF, the number of HF‐related hospitalisations is also rising and any subsequent hospitalization increases the mortality risk of patients with HF.^3^

Ceramides (Cer) are a class of bioactive lipids playing a vital role in many cellular functions and their increased circulating levels have been linked to low‐grade inflammation and cardiometabolic diseases.^4^ Cer containing C16, C18 and C24:1 acyl chains and their ratios have been shown to have a predictive value of CV death in patients with stable CAD and acute coronary syndrome (ACS).^5^ Notably, higher levels of C16 were found to be associated with an increased risk of HF events.^6^ Data from the Framingham Heart study and the Study of Health in Pomerania has reported that a higher C16:0/24:0 Cer ratio was significantly associated with an increased risk of incident HF.^7^ Another study found that higher levels of Cer d18:1/24:0 were associated with an increased risk of CV death in patients with chronic HF.^8^ Therefore, different plasma Cer appear to have a strong prognostic value and can be used as risk factors for assessing cardiac pathology irrespective of traditional plasma lipid markers.

The Cardiovascular Event Risk Test (CERT) risk score, which is based on specific circulating Cer and phospholipid species, has been developed and validated to improve the CV risk prediction in patients with CAD and ACS, and it has been shown to be a superior tool compared to more traditional CV risk markers.^5^ The CERT1 risk score uses distinct plasma Cer and their ratios to assess the risk for major CV events and can be further improved by combining these plasma Cer with phosphatidylcolines (PCs).^5^, ^9^ Superior to the CERT1 risk score as a prognostic marker in several clinical settings, the CERT2 risk score, based on plasma Cer and PCs, has been shown to efficiently identify patients with CAD that are at an increased risk of CV death.^9^ To our knowledge, however, the prognostic value of CERT risk scores has not been evaluated in patients with HF.

Therefore, we used data obtained from the COMMANDER‐HF and GISSI‐HF trials to examine the relationship between distinct plasma Cer and phospholipid species and the risk of developing major adverse CV events.

## 2 METHODS

### 2.1 Ethical approval

COMMANDER HF has been conducted in keeping with Good Clinical Practice guidelines, the principles outlined in the Declaration of Helsinki, and applicable local laws and regulations. All participating centres/countries had to obtain approval from appropriate independent ethics committees or institutional review boards, and patients had to give written informed consent.

The GISSI‐HF trial was approved by each local Institutional Review Board of all the participating centres. A written informed consent was obtained from each study participant before the study enrolment.

### 2.2 Study populations

The COMMANDER‐HF (NCT01877915) study design and main results have been previously described.^10^ The trial randomized 5022 patients with a history of HF and significant CAD following a recent symptomatic exacerbation of HF, 2515 in the placebo group and 2507 in the rivaroxaban group. The median follow‐up was 1.7 years. The present study includes 4234 patients with available baseline samples for CERT risk score analyses, 2129 in the placebo group and 2105 in the rivaroxaban group, respectively. The baseline characteristics of the study population can be found in Table 1.

**TABLE 1:** Baseline characteristics of the study participants.

	COMMANDER‐HF (*n* = 4234)	GISSI‐HF (*n* = 1227)
Age (years)	67 (59–74)	71 (63–78)
Female sex	968 (22.9%)	239 (19.5%)
BMI (kg/m^2^)	27.3 (24.5–30.9)	26.4 (24.0–29.4)
Systolic BP (mmHg)	122 (110–132)	120 (110–140)
Diastolic BP (mmHg)	74 (68–80)	80 (70–80)
Ischemic heart failure	100%	626 (51.0%)
eGFR (mL/min/1.73m^2^)	65.9 (51.4–81.2)	65.8 (49.0–76.1)
eGFR		
<30 mL/min/1.73m^2^	133 (3.1%)	53 (4.3%)
30–59.9 mL/min/1.73m^2^	1546 (36.5%)	483 (39.4%)
60–89.9 mL/min/1.73m^2^	1896 (44.8%)	540 (44.0%)
≥90 mL/min/1.73m^2^	659 (15.6%)	144 (11.7%)
LVEF (%)	34 (28–38)	33 (27–38)
NYHA class		
Class I	132 (3.1%)	0
Class II	1905 (45.0%)	907 (73.9%)
Class III–IV	2196 (51.8%)	320 (26.1%)
Haemoglobin (g/dL)	13.6 (12.4–14.8)	13.7 (12.6–14.7)
Medical history		
History of MI	3293 (77.8%)	539 (43.9%)
History of stroke	385 (9.1%)	55 (4.5%)
History of diabetes	1731 (40.9%)	320 (26.1%)
History of hypertension	3290 (77.7%)	673 (54.8%)
History of PCI/CABG	2659 (62.8%)	369 (30.1%)
Cardiac device	575 (13.6%)	N/A
Cardiac resynchronization therapy	83 (2.0%)	N/A
Implantable cardioverter defibrillator	387 (9.1%)	101 (8.2%)
Pacemaker	183 (4.3%)	173 (14.1%)
Drug therapy		
Diuretic	4221 (99.7%)	1113 (90.7%)
ACEI/ARB	3967 (93.7%)	999 (81.4%)
Nitrate	819 (19.3%)	385 (31.4%)
Hydralazine	45 (1.1%)	N/A
Beta‐blocker	3936 (93.0%)	835 (68.1%)
MRA	3189 (75.3%)	529 (43.1%)
Digoxin	264 (6.2%)	416 (33.9%)
Aspirin	3962 (93.6%)	599 (48.8%)
Thienopyridine	1645 (38.9%)	143 (11.7%)
Antiplatelet therapy		
Aspirin alone	2529 (59.7%)	568 (46.3%)
Thienopyridine alone	212 (5.0%)	112 (9.1%)
Dual antiplatelet	1433 (33.8%)	31 (2.5%)
None	60 (1.4%)	516 (42.1%)

The GISSI‐HF (NCT00336336) trial was a pragmatic, double‐blind, placebo‐controlled, randomized, multicentre study to assess the efficacy and safety of n‐3 polyunsaturated fatty acids [PUFAs] and rosuvastatin in patients with symptomatic chronic HF with any left ventricular ejection fraction. Not all patients had a history of CAD, but an ischemic aetiology (e.g. previous acute myocardial infarction or chronic CAD) was documented in 50% of these. patients. The trial was conducted in 357 Italian centres and enrolled 6975 patients with clinical evidence of chronic and stable HF from August 2002 to February 2005 and obtained permission from ethical commission. The median follow‐up was 3.9 years. Primary endpoints of the trial were time to death, and time to death or admission to hospital for CV reasons.^11^ For 1227 patients baseline samples where available of whom 1063 had samples assessed also at month 3.

### 2.3 End‐point assessment

The primary outcome of interest for this analysis was CV death in association with plasma lipid variables while utilizing the prognostic value of CERT1 and CERT2 risk scores.^9^ Since the CERT2 risk score is the evolution of the CERT1 risk score, the present analysis is mainly focused on CERT2. Additional outcomes of interest for this analysis included all‐cause death and major CV events, that is, a three‐point MACE defined as a composite endpoint of nonfatal myocardial infarction, nonfatal stroke or CV death.

### 2.4 Quantification of ceramides and phosphatidylcholines

The plasma levels of Cer(d18:1/16:0), Cer(d18:1/18:0), Cer(d18:1/24:0), Cer(d18:1/24:1), PCs(14:0/22:6), PCs(16:0/16:0), PCs(16:0/22:5) were quantified on a 5500 QTRAP mass spectrometer equipped with an UHPLC system as described recently.^5^, ^12^ PCs were analysed from the same lipid extract as the Cer.^9^

### 2.5 CERT2 risk score

The CERT2 risk score comprises distinct plasma Cer and PCs lipid ratios combined with a single PCs:

- Cer(d18:1/24:1)/Cer(d18:1/24:0),
- Cer(d18:1/16:0)/PCs(16:0/22:5),
- Cer(d18:1/18:0)/PCs(14:0/22:6),
- PCs(16:0/16:0)

Patients were assigned a score between 0 and 3 (CERT2) for quartiles 1–4 respectively as specified in Table 2. A patient's CERT2 risk score was then calculated by adding all four components to create a score ranging from 0 to 12, subsequently stratified into four risk categories ranging from low to high risk. More details of the CERT2 risk score calculation have been previously described.^9^

**TABLE 2:** CERT2 risk score assigned based on plasma Cer and PCs lipid ratios for quartiles (Q) 1–4.

CERT2	Q1	Q2	Q3	Q4	Score
Cer(d18:1/24:1)/Cer(d18:1/24:0)	0	1	2	3	0–12
Cer(d18:1/16:0)/PCs(16:0/22:5)	0	1	2	3	
Cer(d18:1/18:0)/PCs(14:0/22:6)	0	1	2	3	
PCs(16:0/16:0)	0	1	2	3	

### 2.6 Statistical analyses

Comparison of baseline characteristics and distinct lipid variables (i.e. lipids, lipid ratios and CERT2 score) between patients allocated to active treatment (i.e. rivaroxaban or n‐3PUFA) and placebo was carried out using the non‐parametric Wilcoxon test for continuous variables and the Fisher's exact test for categorical variables. Continuous variables were expressed as median (interquartile range) and categorical variables as frequency (percentage). No differences were observed by study treatment groups at baseline, and therefore, within the two study populations, patients were treated as a single group. All analyses in the GISSI‐HF trial were adjusted by study treatments when applied to changes after 3‐month follow‐up.

To assess the association between each lipid variable and each study endpoint (CV death, all‐cause death or three‐point MACE), time‐to‐event analyses were performed using three progressive Cox regression models adjusted as follows: model 1: unadjusted; model 2: adjusted for age and sex; model 3: adjusted for age, sex, body mass index, arterial hypertension, diabetes, left ventricular ejection fraction (LVEF), estimated glomerular filtration rate (eGFR, calculated with the simplified Modification of Diet in Renal Disease (MDRD) equation), and treatment group; model 4: model 3 + adjustment for Ln‐transformed NT‐proBNP. Hazard ratios (HRs) are presented with their 95% confidence intervals (95% CI). Cox models were used to assess interactions between lipid variables (quartiles for lipids and lipid ratios, and risk groups for CERT2 score) and the study treatment. HR of study treatment vs. placebo in each subgroup was reported (HR > 1: placebo was better, HR < 1: study/treatment was better). Changes of CERT2 over 3 months in the GISSI‐HF trial were calculated as relative to the value at randomization and compared using the Mann–Whitney test between treatment groups.

Cumulative events for CV death, all‐cause death and three‐point MACE according to lipid groups (quartiles for lipids and lipid ratios, CERT2 risk score groups) were illustrated using the Kaplan–Meier analyses. Difference between survival curves were analysed using the log‐rank test. Kaplan–Meier analyses were used to show the added value of CERT2 highest scores 9–12 on top of NT‐proBNP higher than 450 pg/mL (upper limit of first tertile). This was possible only for the GISSI‐HF cohort, since NT‐proBNP had been assayed.

All statistical analyses were performed using R software (the R foundation for Statistical Computing). The two‐tailed significance level was set at *p*‐value < 0.05.

## 3 RESULTS

### 3.1 CERT2 risk score, all‐cause death and cardiovascular endpoints

**FIGURE 1:**
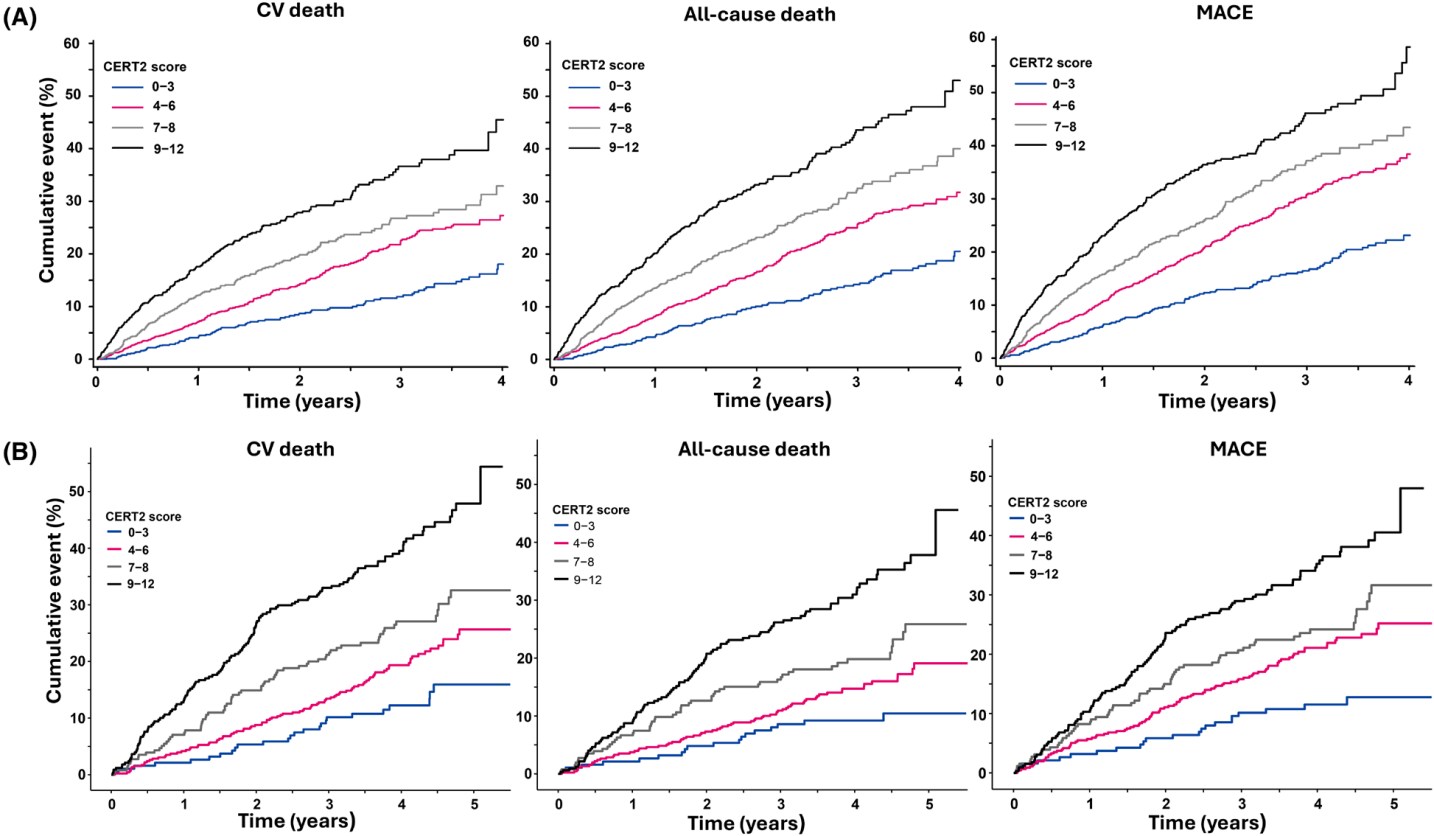
Kaplan–Meier curves of the progressively increased risks for CV death, all‐cause death and MACE.

The performance of the CERT2 risk score was evaluated for CV death, all‐cause death and three‐point MACE. In the unadjusted risk of CV death in the COMMANDER‐HF trial (Table 3A), an increase was observed across increasing CERT2 risk score categories with more than a three‐fold higher risk of CV death observed for patients in the highest CERT2 risk group (unadjusted HR 3.56, 95% CI 2.78–4.56). In the unadjusted risk of all‐cause death, a slightly higher increase was observed compared to CV death for the CERT2 risk score (unadjusted HR 3.82, 95% CI 3.04–4.80). In the unadjusted risk for MACE, the same trend was observed, an increase across increasing CERT2 risk categories (unadjusted HR 3.46, 95% CI 2.80–4.28). Similar results were found in the GISSI‐HF trial: for all three endpoints, risks increased significantly along with the CERT2 risk score (Table 3B). The progressively increased risks for CV death, all‐cause death and MACE are also visualized in the Kaplan–Meier curves in Figure 1. Upon including a correction for NT‐proBNP in model 4, CERT2 9–12 independently predicted all cause death (HR 1.79, 95% CI 1.14–2.81) and MACE (HR 1.62, 95% CI 1.00–2.63).

**TABLE 3A:** Associations of CERT2 risk score with all‐cause death and cardiovascular endpoints in Cox models for the COMMANDER‐HF participants.

		*n*_event_/*n* (%)	Model 1		Model 2		Model 3	
			HR (CI 95%)	*p*‐value	HR (CI 95%)	*p*‐value	HR (CI 95%)	*p*‐value
CV death	CERT2 score							
	0–3	88/825 (10.7)	Reference	–	Reference	–	Reference	–
	4–6	262/1656 (15.8)	1.73 (1.35–2.20)	<0.0001	1.67 (1.31–2.13)	<0.0001	1.49 (1.17–1.90)	0.001
	7–8	183/921 (19.9)	2.35 (1.82–3.03)	<0.0001	2.26 (1.75–2.91)	<0.0001	1.89 (1.46–2.45)	<0.0001
	9–12	227/832 (27.3)	3.56 (2.78–4.56)	<0.0001	3.37 (2.63–4.32)	<0.0001	2.80 (2.18–3.60)	<0.0001
All‐cause death	CERT2 score							
	0–3	102/825 (12.4%)	Reference	–	Reference	–	Reference	–
	4–6	308/1656 (18.6%)	1.76 (1.41–2.21)	<0.0001	1.71 (1.36–2.13)	<0.0001	1.51 (1.21–1.90)	0.0003
	7–8	224/921 (24.3%)	2.51 (1.98–3.17)	<0.0001	2.40 (1.90–3.04)	<0.0001	2.00 (1.58–2.54)	<0.0001
	9–12	278/832 (33.4%)	3.82 (3.04–4.80)	<0.0001	3.59 (2.85–4.51)	<0.0001	2.97 (2.36–3.75)	<0.0001
3‐point MACE	CERT2 score							
	0–3	121/825 (14.7%)	Reference	–	Reference	–	Reference	–
	4–6	378/1656 (22.8%)	1.84 (1.50–2.25)	<0.0001	1.78 (1.45–2.18)	<0.0001	1.60 (1.30–1.97)	<0.0001
	7–8	253/921 (27.5%)	2.41 (1.94–2.99)	<0.0001	2.31 (1.86–2.87)	<0.0001	1.95 (1.56–2.43)	<0.0001
	9–12	298/832 (35.8%)	3.46 (2.80–4.28)	<0.0001	3.25 (2.63–4.02)	<0.0001	2.73 (2.20–3.38)	<0.0001

**TABLE 3B:** Association of CERT2 risk score with all‐cause death and cardiovascular endpoints in Cox models for the GISSI‐HF participants.

		*n*_event_/*n* (%)	Model 1		Model 2		Model 3		Model 4	
			HR (CI 95%)	*p*‐value	HR (CI 95%)	*p*‐value	HR (CI 95%)	*p*‐value	HR (CI95%)	*p*‐value
CV death	CERT2 score									
	0–3	18/188	Reference	–	Reference	–	Reference	–	Reference	–
	4–6	70/455	1.66 (0.99–2.79)	0.055	1.52 (0.91–2.56)	0.112	1.45 (0.86–2.44)	0.162	1.20 (0.71–2.03)	0.491
	7–8	53/255	2.39 (1.40–4.07)	**0.001**	2.01 (1.17–3.45)	**0.011**	1.78 (1.04–3.06)	**0.036**	1.30 (0.75–2.26)	0.345
	9–12	101/329	4.00 (2.42–6.61)	**<0.0001**	3.10 (1.86–5.16)	**<0.0001**	2.84 (1.70–4.74)	**<0.0001**	1.60 (0.94–2.71)	0.083
All‐cause death	CERT2 score									
	0–3	25/188	Reference	–	Reference	–	Reference	–	Reference	–
	4–6	95/455	1.63 (1.05–2.53)	**0.031**	1.48 (0.95–2.30)	0.082	1.43 (0.92–2.23)	0.110	1.24 (0.80–1.93)	0.344
	7–8	71/255	2.31 (1.47–3.65)	**<0.0001**	1.93 (1.22–3.05)	**0.005**	1.78 (1.12–2.82)	**0.015**	1.39 (0.87–2.21)	0.169
	9–12	137/329	3.95 (2.58–6.05)	**<0.0001**	3.00 (1.96–4.64)	**<0.0001**	2.83 (1.83–4.38)	**<0.0001**	1.79 (1.14–2.81)	**0.011**
3‐point MACE	CERT2 score									
	0–3	22/188	Reference	–	Reference	–	Reference	–	Reference	–
	4–6	97/455	1.94 (1.22–3.07)	**0.005**	1.81 (1.14–2.88)	**0.012**	1.73 (1.09–2.76)	**0.020**	1.48 (0.93–2.67)	0.098
	7–8	65/255	2.43 (1.50–3.95)	**<0.0001**	2.10 (1.29–3.42)	**0.003**	1.86 (1.14–3.04)	**0.013**	1.45 (0.88–2.37)	0.145
	9–12	111/329	3.63 (2.30–5.74)	**<0.0001**	2.90 (1.82–4.62)	**<0.0001**	2.67 (1.67–4.26)	**<0.0001**	1.62 (1.00–2.63)	**0.048**

**FIGURE 2:**
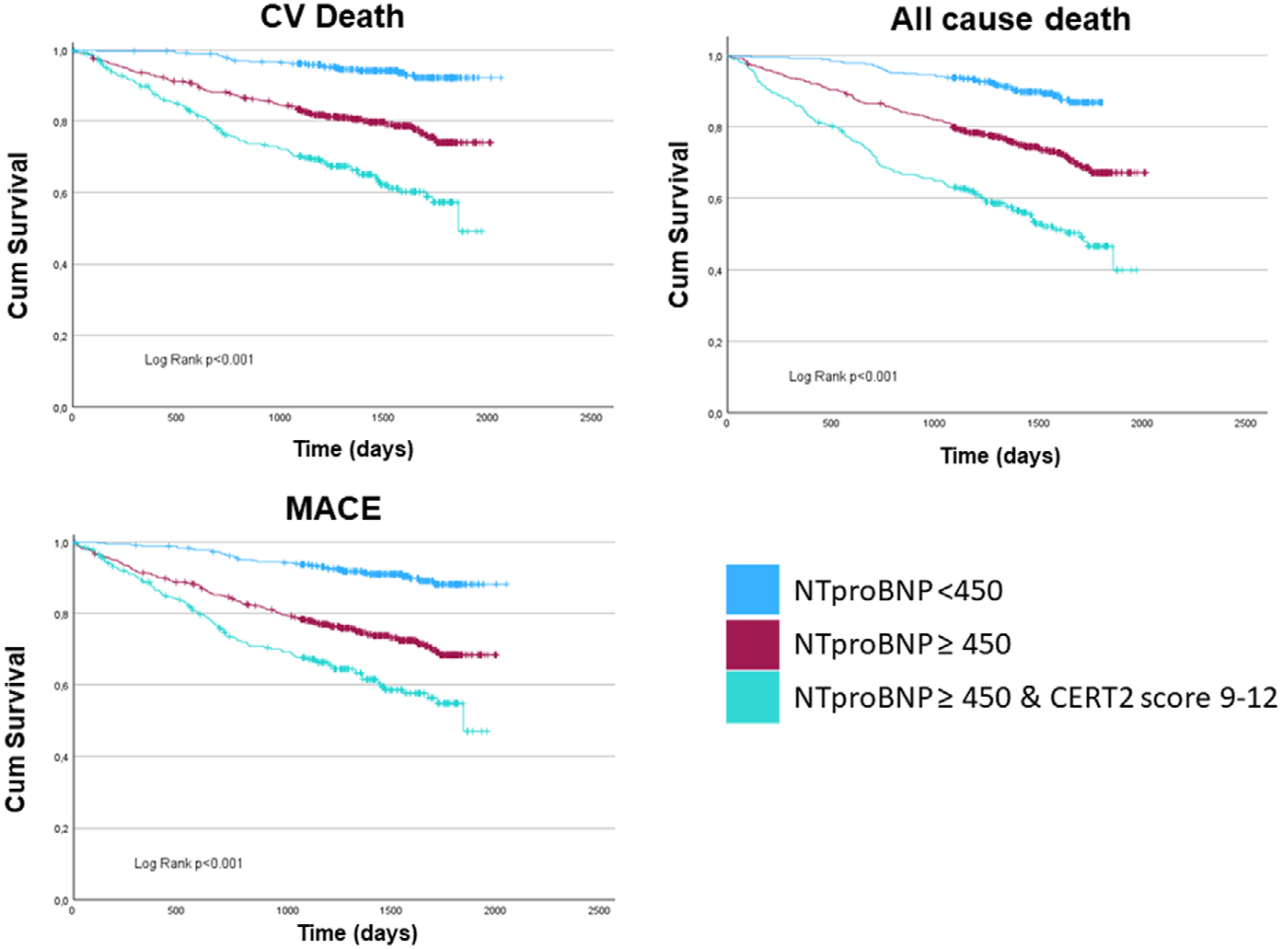
Kaplan–Meier curves of NT‐proBNP for CV death, all‐cause death and MACE.

Overall, in both studies the higher CERT2 risk score category remained significantly associated with a greater risk of CV death, all‐cause death and three‐point MACE after adjusting for age and sex (model 2). After further adjustment for other CV risk factors and potential confounders (age, sex, BMI, hypertension, diabetes, LVEF, eGFR and treatment allocation) Rivaroxaban (COMMANDER‐HF) or n‐3PUFA (GISSI‐HF), adjusted model 3), a significant association remained between the highest CERT2 risk score category and the risk of CV death (COMMANDER‐HF: HR 2.80, 95% CI 2.18–3.60; GISSI‐HF: 2.84, 95% CI 1.70–4.74). Same results for all‐cause death (COMMANDER‐HF: HR 2.97, 95% CI 2.36–3.75; GISSI‐HF: 2.83, 95% CI 1.83–4.38) and MACE (COMMANDER‐HF: HR 2.73, 95% CI 2.20–3.38; GISSI‐HF: 2.67, 95% CI 1.67–4.26). In the GISSI‐HF cohort, when Ln‐transformed NT‐proBNP was included, a significant association was observed with all‐cause death (HR 1.79, 95% CI 1.14–2.81) and MACE (HR 1.62, 95% CI 1.00–2.63) for individuals in the highest CERT2 risk score category. The added value of CERT2 was evident from univariate Kaplan–Meier curves (Figure 2).

Upon performing this analysis in the subgroup of 626 GISSI‐HF participants with HF of ischemic aetiology, similar significant results were found for the CERT2 risk score, despite a lower number of patients included in this analysis, see Table S1.

### 3.2 Lipid species level association with all‐cause death and cardiovascular endpoints

The prognostic performance of different plasma Cer/PCs species and their distinct ratios was also explored (Table S2A,B). Robust associations were recorded between the prespecified lipid ratios and the risk of all study endpoints, both before and after multivariable adjustments.

### 3.3 Standard lipid variable association with all‐cause death and cardiovascular endpoints

For the GISSI‐HF trial, the following standard plasma lipid variables were measured locally: total cholesterol, HDL‐cholesterol, LDL‐cholesterol, and triglycerides allowing comparison with Cer and phospholipid species (Table 4). In the unadjusted risk for CV death and all‐cause death, all four lipids have shown a significant inverse association opposite to the positive association between the CERT2 risk score and the risk of CV outcomes. After adjusting for age and sex (model 2), these associations remained statistically significant. However, after further adjustment for other covariates (age, sex, BMI, hypertension, diabetes, LVEF, eGFR and treatment allocation, model 3), the significance of associations tended to attenuate and remained significant only for plasma total cholesterol and triglyceride concentrations. Similar lipid associations were also observed for the three‐point MACE.

**TABLE 4:** Associations of standard lipid variables with all‐cause death and cardiovascular endpoints in Cox models for the GISSI‐HF participants.

	*n*_event_/*n*	Model 1		Model 2		Model 3	
		HR (CI 95%)	*p*‐value	HR (CI 95%)	*p*‐value	HR (CI 95%)	*p*‐value
Total cholesterol							
CV death	241/1222	0.73 (0.65–0.82)	<0.0001	0.68 (0.58–0.79)	<0.0001	0.50 (0.26–0‐96)	0.038
All cause death	327/1222	0.74 (0.66–0.82)	<0.0001	0.68 (0.59–0.78)	<0.0001	0.46 (0.27–0.78)	0.004
3‐point MACE	607/1222	0.84 (0.78–0.91)	<0.0001	0.85 (0.76–0.95)	0.005	1.15 (0.56–2.33)	0.708
LDL‐C							
CV death	217/1134	0.82 (0.73–0.92)	<0.0001	0.82 (0.70–0.97)	0.019	1.20 (0.87–1.66)	0.268
All cause death	295/1134	0.83 (0.75–0.92)	<0.0001	0.84 (0.73–0.98)	0.021	1.27 (0.95–1.70)	0.106
3‐point MACE	561/1134	0.86 (0.80–0.93)	<0.0001	0.87 (0.78–0.97)	0.016	1.05 (0.86–1.28)	0.632
HDL‐C							
CV death	235/1204	0.82 (0.72–0.94)	0.003	0.79 (0.66–0.95)	0.011	0.96 (0.79–1.16)	0.671
All cause death	319/1204	0.88 (0.79–0.99)	0.029	0.78 (0.67–0.91)	0.002	0.94 (0.80–1.11)	0.485
3‐point MACE	597/1204	0.89 (0.82–0.96)	0.005	0.86 (0.76–0.96)	0.007	0.92 (0.81–1.05)	0.207
Triglycerides							
CV death	240/1218	0.82 (0.72–0.94)	0.004	0.76 (0.63–0.91)	0.003	0.79 (0.64–0.98)	0.030
All cause death	326/1218	0.80 (0.71–0.90)	<0.0001	0.76 (0.65–0.89)	<0.0001	0.81 (0.67–0.97)	0.019
3‐point MACE	605/1218	0.94 (0.87–1.02)	0.142	0.98 (0.87–1.10)	0.719	1.01 (0.89–1.14)	0.937

We have assessed the change over 3 months of the CERT2 score by treatment with n‐3PUFA or placebo in GISSI‐HF. While no differences in CERT2 score were found at baseline, after 3 months of n‐3PUFA intake the CERT2 scores were reduced with 15% as compared to patients who were randomized to placebo (*p* < 0.001), see Table 5.

**TABLE 5:** CERT2 scores in placebo and n‐3PUFA groups after 3 months of treatment in GISSI‐HF.

CERT2 score	Placebo (*n* = 610)	n‐3PUFA (*n* = 617)	Relative difference (%)**	*p*‐value*
Baseline	6 (5–9)	6 (4–9)	−3.19	0.155
3 months	6 (4–8)	5 (3–7)	−14.99	1.0 × 10^−7^

*Note*: Values are median (Q1–Q3).

* Mann–Whitney test for difference between treatment groups.

** Relative difference is for the means with placebo as reference.

## 4 DISCUSSION

The findings of the present large study demonstrate that a plasma Cer and phospholipid‐based score efficiently identifies patients with HF who are at higher risk of CV death. The CERT2 risk score can also be used to identify those patients who are at higher risk of all‐cause death and three‐point MACE (defined as nonfatal myocardial infarction, nonfatal stroke and CV mortality). Therefore, CERT2 provides incremental information for risk stratification of patients with HF. Notably, independent analyses were done in the plasma samples of the two cohorts of patients from the COMMANDER‐HF and GISSI‐HF trials. We believe that this increases the strength and generalizability of our results.

It is noteworthy that despite the very high event rate in the COMMANDER‐HF trial, the CERT2 risk score enabled a clear risk stratification in the HF patient population. In fact, patients with the highest CERT2 risk score had over 3.5‐fold increased risk for CV death, 3.8‐fold increased risk for all‐cause death and 3.4‐fold increased risk for three‐point MACE compared to those with the lowest CERT2 risk score. After adjusting for important clinical variables, such as age and sex, there still was an almost three‐fold increased risk of death for patients at the highest CERT2 risk score.

Whenever a new biomarker of risk is tested in HF, its added value on top of the benchmark of a natriuretic peptide has to be assessed. When the model was adjusted for the benchmark biomarker NT‐proBNP, CERT2 was no longer independently related to CV‐death and it's relation with MACE was of borderline significance, while a strong association for Ln‐transformed NT‐proBNP could be observed: 1.95 (1.69–2.26) and 1.76 (1.55–2.00), respectively for CV death and MACE. Nonetheless CERT2 independently predicted all‐cause death even with a statistically significant adjustment for NT‐proBNP which had a HR of 1.71 (1.51–1.93). The two markers of risk may reflect different pathophysiological pathways. which confirms the prognostic value of NT‐proBNP for cardiac events in HF.

The CERT2 risk score has been previously validated as an important tool for predicting CV events in patients with CAD.^5^ The present study provides clear evidence that the CERT2 risk score can also be used as a prognostic biomarker in addition to established biomarkers of outcome in patients with HF or CAD.

Notably, the results were replicated in the GISSI‐HF study, which showed similar results to those of the COMMANDER‐HF trial, both in univariate analyses and even after adjusting for age and sex. It is important to note the two cohorts included patients with HF, however, in the COMMANDER‐HF trial all patients had LVEF ≤ 40%, CAD and a recent episode of worsening of HF, while in the GISSI‐HF trial all patients had symptomatic HF of any LVEF and about half of them with ischemic aetiology of HF. Furthermore, the cumulative rates of all‐cause mortality in the COMMANDER‐HF and GISSI‐HF trials were 22% over a median of 1.7 years and 28% over a median of 3.9 years, respectively.

As previously reported, while in the COMMANDER‐HF trial all patients had HF of ischemic aetiology, only half of those included in the GISSI‐HF trial had an ischemic aetiology of HF. That said, the aetiology of HF in the GISSI‐HF participants did not influence either the prognostic value or the changes of CERT2 risk score over time. These apparently unexpected findings from the GISSI‐HF trial may suggest that the CERT2, a score specifically developed and targeted to estimate the mortality risk in CAD,^9^ may lose, at least in part, its specificity in the context of chronic HF. It may well be that overt HF overrides lipid and inflammatory pathways in predicting patient outcomes.

Various plasma Cer and PCs species are potential risk markers in different pathologies. Long and very‐long‐chain plasma Cer appear to be the main contributors to an increased risk of CV death in patients with chronic HF.^8^ A strong association between Cer(d18:1/18:0) and risk of MACE has been previously reported.^13^ Our data supports a strong association between distinct plasma Cer species and increased risk of MACE in patients with HF. An increased Cer ratio of Cer(d18:1/16:0)/Cer(d18:1/24:0) has been previously found to be strongly associated with the risk of CV death in patients with ACS and CAD.^5^ An increased Cer(d18:1/16:0)/Cer(d18:1/24:0) ratio is also significantly associated with a higher risk of CV death in patients with HF, as can be seen in our study. Moreover, the Cer(d18:1/16:0)/Cer(d18:1/24:0) ratio shows a strong association with risks of all‐cause death and MACE. Another study found that increased levels of Cer(d18:1/24:1) and the ratios Cer(d18:1/16:0)/Cer(d18:1/24:0), Cer(d18:1/18:0)/Cer(d18:1/24:0), Cer(d18:1/24:1)/Cer(d18:1/24:0) and Cer(d18:1/16:0)/PCs(16:0/22:5) are strongly associated with a higher risk of MACE.^14^ Numerous studies have explored specific plasma Cer and PCs, and their involvement in the increased risk of adverse CV outcomes. Our data contributes to the importance of utilizing plasma lipid ratios and Cer risk scores in identifying patients with cardio metabolic diseases at high risk for major CV events.

Evolving research underscores the linkage between Cer lipids and CVD risk, shedding light on the molecular mechanisms underlying the pathogenesis of CV disorders.^15^ Cer have gained attention due to their multifaceted involvement in cellular processes that contribute to CV health.^4^ These lipids, known for their role in membrane structure and signalling pathways, exhibit pro‐inflammatory properties, promoting endothelial dysfunction and vascular inflammation. The impact of Cer extends beyond inflammation, encompassing oxidative stress and apoptosis, processes intricately linked to the progression of CVD. Cer have also been implicated in insulin resistance further amplifying CVD risk. Finally, through their influence on lipid metabolism and cellular signalling cascades, Cer contribute to a milieu conducive to atherosclerosis and plaque formation.^16^, ^17^

Standard lipid variables have been suggested to have prognostic value in predicting CV events.^18^ In the current study, a standard lipid panel was measured allowing comparison between routinely used plasma lipids and CERT2 risk score as well as its Cer components. Opposite results were observed between standard lipids and Cer species, Cer/phospholipid ratios and CERT2 risk score. Interestingly, it appeared that while the CERT2 risk score and its Cer components were positively associated with the risk of CV outcomes, the routinely used lipids, such as plasma total cholesterol and LDL‐C concentrations, had an unexpected inverse association with CV events during the follow‐up. This observation also supports earlier studies demonstrating a lack of association between plasma LDL‐C concentration and the risk of CV events in elderly or patients with established CAD.^19^, ^20^ Based on the present results, it appears that plasma LDL‐C concentration cannot be used as a reliable CV risk marker in people with chronic HF, despite its causal role in the development of atherosclerosis. However, as most circulating Cer are transported in LDL particles, they remain of clinical interest as their manipulation might be of therapeutic value.

The present study has some limitations. The most important is the heterogeneity between the two populations: all patients in COMMANDER‐HF had documented ischemic heart disease (IHD), while GISSI‐HF included unselected HF patients. However, adjusting for the trial factor did not diminish the prognostic value of CERT2 in the multivariable analysis. NT‐proBNP was not measured in COMMANDER‐HF, so we were only able to evaluate the added prognostic value of CERT2 alongside NT‐proBNP in the 1227 patients from GISSI‐HF. Lastly, the GISSI‐HF samples had been stored for approximately 20 years at −70°C in an ISO 9001:2015 certified biobank, part of BBMRI, with continuous temperature monitoring. Despite the long storage time, the absolute concentrations of the six components of the CERT2 score were well within the expected ranges.

## 5 CONCLUSIONS

The strength of the present analysis is that two independent large cohorts of patients with HF with slightly different characteristics were studied, and in both patient cohorts, the prognostic value of the CERT2 risk score showed superimposable trends. Sphingolipids and Cer were assayed in plasma with the same analytical methods. The apparent differences in risk estimates between the two patient cohorts cannot be addressed adequately since the GISSI‐HF cohort of 1227 patients is much smaller than that of the COMMANDER‐HF cohort of 4234 patients.

In conclusion, in this study of patients with a history of HF and significant CAD we found for the first time that plasma Cer‐phospholipid risk score (i.e. the CERT2 risk score) was significantly associated with an increased risk of CV death, all‐cause death and three‐point MACE independent of traditional CV risk factors and risk markers. This risk score provides significant predictive value in risk stratification and can improve the prediction of major CV events, including death in people with HF, most with significant CAD. Combining the CERT2 risk score and other risk stratification methods might further improve the identification of patients at an increased risk of developing major CV events.

### 5.1 Clinical perspectives

The CERT2 risk score provides significant predictive value in risk stratification and improves the prediction of major CV events. Combining the CERT2 risk score with other risk stratification can be a tool for clinicians to identify patients at an increased risk of developing major CV events.

## FUNDING INFORMATION

This work has been supported by Fondazione Regionale per la Ricerca Biomedica (Regione Lombardia), Project ERAPERMED2022‐113, GA 779282. This project has also received funding from the European Union's Horizon 2020 research and innovation programme under the grant agreement No. 848056 (CoroPrevention) and Horizon Europe grant agreement No. 101095413 (CARE‐IN‐HEALTH). Views and opinions expressed are those of the author(s) only and do not necessarily reflect those of the European Union or the European Commission. Neither the European Union nor the granting authority can be held responsible for them.

## CONFLICT OF INTEREST STATEMENT

Zora Biosciences Oy holds patent disclosures related to the diagnostic and prognostic use of ceramides and phospholipids in CVD. M.H., A.J., and R.L. are employees and R.L. a shareholder of Zora Biosciences Oy. All other authors have nothing to disclose. This work has been supported by Fondazione Regionale per la Ricerca Biomedica (Regione Lombardia), project ERAPERMED2022‐113, GA779282 under the frame of ERA PerMed. The GISSI‐HF clinical trial was funded by Società Prodotti Antibiotici (SPA; Italy), Pfizer, Sigma Tau, and AstraZeneca.

## Supporting information


Data S1.

## Data Availability

The data that support the findings of this study are available on request from the corresponding author, JMTAM. The data are not publicly available due to containing information that could compromise the privacy of research participants.
