# Supplementary material for: Ceramide and phosphatidylcholine lipids‐based risk score predicts major cardiovascular outcomes in patients with heart failure
Source: Eur J Clin Invest. 2024 Nov 23;55(3):e14359. doi: 10.1111/eci.14359 (PMC11810540; doi:10.1111/eci.14359)
Supplement: Supplementary file 1 — Data S1. [file ECI-55-e14359-s001.docx]

**SUPPLEMENTARY MATERIAL**

**CERT2 risk score predicts major cardiovascular outcomes in patients with heart failure**

Angelika Witoslawska MSc^1^, Jennifer M.T.A. Meessen PhD^2^, Mika Hilvo PhD ^3,4^, Antti Jylhä^3^, Faiez Zannad Prof^5^, Marianna Cerrato MSc^2^, Patrick Rossignol Prof^5^, Deborah Novelli PhD^2^, Kevin Duarte PhD^5^, Giovanni Targher Prof^6,7^, Roberto Latini MD^2^, Nicolas Girerd Prof^5^, Reijo Laaksonen PhD ^1,3^

1. Finnish Cardiovascular Research Center, University of Tampere, Tampere, Finland
2. Department of Acute Brain and Cardiovascular Injury, Institute for Pharmacological Research Mario Negri IRCCS, Milan, Italy.
3. Zora Biosciences Oy, Espoo Finland
4. VTT Technical Research Centre of Finland Ltd
5. Centre d'Investigation Clinique Plurithémathique Pierre Drouin & Département de Cardiologie, Institut Lorrain du Coeur et des Vaisseaux, CHU de Nancy, France
6. Department of Medicine, University of Verona, Italy
7. Metabolic Diseases Research Unit, IRCCS Sacro Cuore - Don Calabria Hospital, Negrar di Valpolicella (VR), Italy

**Table S1.** Associations of continuous CERT2 score with all-cause death and cardiovascular endpoints in Cox models for GISSI-HF participants with HF (n=626).

|  | | **Model 1** | | **Model 2** | | **Model 3** | | **Model 4** | |
| --- | --- | --- | --- | --- | --- | --- | --- | --- | --- |
|  |  | **HR (CI 95 %)** | **p-value** | **HR (CI 95 %)** | **p-value** | **HR (CI 95 %)** | **p-value** | **HR (CI 95 %)** | **p-value** |
| CV death | All | 1.21 (1.15-1.26) | <0.0001 | 1.17 (1.10-1.22) | <0.0001 | 1.16 (1.10-1.22) | <0.0001 | 1.07 (1.01-1.12) | 0.013 |
|  | Ischemic | 1.17 (1.10-1.24) | <0.0001 | 1.13 (1.06-1.20) | 0.0003 | 1.12 (1.05-1.20) | 0.0009 | 1.04 (0.97-1.12) | 0.276 |
| All-cause death | All | 1.20 (1.16-1.25) | <0.0001 | 1.16 (1.11-1.21) | <0.0001 | 1.16 (1.11-1.21) | <0.0001 | 1.08 (1.04-1.13) | 0.0004 |
|  | Ischemic | 1.17 (1.11-1.24) | <0.0001 | 1.14 (1.08-1.20) | <0.0001 | 1.13 (1.07-1.20) | <0.0001 | 1.07 (1.01-1.14) | 0.026 |
| MACE | All | 1.17 (1.12-1.22) | <0.0001 | 1.13 (1.08-1.18) | <0.0001 | 1.12 (1.07-1.17) | <0.0001 | 1.05 (1.00-1.09) | 0.063 |
|  | Ischemic | 1.14 (1.07-1.20) | <0.0001 | 1.11 (1.04-1.17) | 0.0007 | 1.09 (1.03-1.16) | 0.003 | 1.02 (0.96-1.09) | 0.521 |

**^^[[1]](#footnote-1)^^**

**Table S2A.** Associations of lipid variables with CV-death, all-cause death and 3-point MACE in Cox models in COMMANDER-HF participants.

|  | **n_event_/n (%)** | **Model 1** | | **Model 2** | | **Model 3** | |
| --- | --- | --- | --- | --- | --- | --- | --- |
|  |  | **HR (CI 95 %)** | **p-value** | **HR (CI 95 %)** | **p-value** | **HR (CI 95 %)** | **p-value** |
| **CV death** | | | | | | | |
| Cer(d18:1/16:0) | 760/4234 (17.9%) | 1.18 (1.11 - 1.26) | <0.0001 | 1.19 (1.11 - 1.27) | <0.0001 | 1.17 (1.09 - 1.25) | <0.0001 |
| Cer(d18:1/18:0) | 760/4234 (17.9%) | 1.06 (0.99 - 1.14) | 0.094 | 1.09 (1.01 - 1.17) | 0.023 | 1.11 (1.03 - 1.20) | 0.004 |
| Cer(d18:1/24:0) | 760/4234 (17.9%) | 0.79 (0.73 - 0.84) | <0.0001 | 0.81 (0.75 - 0.87) | <0.0001 | 0.87 (0.80 - 0.93) | 0.0001 |
| Cer(d18:1/24:1) | 760/4234 (17.9%) | 1.12 (1.04 - 1.20) | 0.002 | 1.11 (1.03 - 1.19) | 0.007 | 1.07 (0.99 - 1.15) | 0.082 |
| PC(14:0/22:6) | 760/4234 (17.9%) | 0.77 (0.72 - 0.82) | <0.0001 | 0.76 (0.72 - 0.81) | <0.0001 | 0.79 (0.74 - 0.84) | <0.0001 |
| PC(16:0/16:0) | 760/4234 (17.9%) | 1.07 (1.01 - 1.13) | 0.021 | 1.05 (0.99 - 1.11) | 0.085 | 1.01 (0.95 - 1.08) | 0.71 |
| PC(16:0/22:5) | 760/4234 (17.9%) | 0.84 (0.78 - 0.90) | <0.0001 | 0.83 (0.77 - 0.90) | <0.0001 | 0.84 (0.78 - 0.90) | <0.0001 |
| Cer(d18:1/24:1)/ Cer(d18:1/24:0) | 760/4234 (17.9%) | 1.41 (1.31 - 1.51) | <0.0001 | 1.37 (1.27 - 1.47) | <0.0001 | 1.24 (1.15 - 1.34) | <0.0001 |
| Cer(d18:1/16:0)/  PC(16:0/22:5) | 760/4234 (17.9%) | 1.28 (1.21 - 1.35) | <0.0001 | 1.29 (1.22 - 1.36) | <0.0001 | 1.28 (1.20 - 1.35) | <0.0001 |
| Cer(d18:1/18:0)/  PC(14:0/22:6) | 760/4234 (17.9%) | 1.30 (1.22 - 1.39) | <0.0001 | 1.31 (1.23 - 1.40) | <0.0001 | 1.29 (1.21 - 1.38) | <0.0001 |
| **All-cause death** |  |  |  |  |  |  |  |
| Cer(d18:1/16:0) | 912/4234 (21.5%) | 1.20 (1.13 - 1.27) | <0.0001 | 1.20 (1.13 - 1.27) | <0.0001 | 1.18 (1.11 - 1.25) | <0.0001 |
| Cer(d18:1/18:0) | 912/4234 (21.5%) | 1.07 (1.00 - 1.14) | 0.035 | 1.10 (1.03 - 1.17) | 0.005 | 1.13 (1.06 - 1.20) | 0.0004 |
| Cer(d18:1/24:0) | 912/4234 (21.5%) | 0.78 (0.73 - 0.83) | <0.0001 | 0.80 (0.75 - 0.86) | <0.0001 | 0.86 (0.80 - 0.92) | <0.0001 |
| Cer(d18:1/24:1) | 912/4234 (21.5%) | 1.15 (1.08 - 1.23) | <0.0001 | 1.13 (1.06 - 1.21) | 0.0002 | 1.09 (1.02 - 1.17) | 0.010 |
| PC(14:0/22:6) | 912/4234 (21.5%) | 0.78 (0.74 - 0.82) | <0.0001 | 0.78 (0.73 - 0.82) | <0.0001 | 0.80 (0.75 - 0.84) | <0.0001 |
| PC(16:0/16:0) | 912/4234 (21.5%) | 1.08 (1.03 - 1.14) | 0.0009 | 1.07 (1.02 - 1.12) | 0.010 | 1.03 (0.97 - 1.08) | 0.32 |
| PC(16:0/22:5) | 912/4234 (21.5%) | 0.84 (0.78 - 0.90) | <0.0001 | 0.83 (0.78 - 0.89) | <0.0001 | 0.84 (0.78 - 0.90) | <0.0001 |
| Cer(d18:1/24:1)/  Cer(d18:1/24:0) | 912/4234 (21.5%) | 1.46 (1.37 - 1.55) | <0.0001 | 1.41 (1.32 - 1.50) | <0.0001 | 1.28 (1.19 - 1.37) | <0.0001 |
| Cer(d18:1/16:0)/  PC(16:0/22:5) | 912/4234 (21.5%) | 1.29 (1.23 - 1.36) | <0.0001 | 1.30 (1.24 - 1.36) | <0.0001 | 1.28 (1.22 - 1.36) | <0.0001 |
| Cer(d18:1/18:0)/  PC(14:0/22:6) | 912/4234 (21.5%) | 1.28 (1.21 - 1.36) | <0.0001 | 1.29 (1.22 - 1.37) | <0.0001 | 1.28 (1.21 - 1.36) | <0.0001 |
| **3-point MACE** |  |  |  |  |  |  |  |
| Cer(d18:1/16:0) | 1050/4234 (24.8%) | 1.19 (1.12 - 1.25) | <0.0001 | 1.19 (1.12 - 1.25) | <0.0001 | 1.16 (1.10 - 1.23) | <0.0001 |
| Cer(d18:1/18:0) | 1050/4234 (24.8%) | 1.07 (1.01 - 1.14) | 0.020 | 1.10 (1.03 - 1.16) | 0.003 | 1.12 (1.05 - 1.19) | 0.0003 |
| Cer(d18:1/24:0) | 1050/4234 (24.8%) | 0.81 (0.76 - 0.86) | <0.0001 | 0.83 (0.78 - 0.88) | <0.0001 | 0.88 (0.83 - 0.94) | <0.0001 |
| Cer(d18:1/24:1) | 1050/4234 (24.8%) | 1.16 (1.09 - 1.23) | <0.0001 | 1.14 (1.07 - 1.21) | <0.0001 | 1.10 (1.03 - 1.17) | 0.003 |
| PC(14:0/22:6) | 1050/4234 (24.8%) | 0.81 (0.77 - 0.86) | <0.0001 | 0.81 (0.77 - 0.86) | <0.0001 | 0.83 (0.79 - 0.88) | <0.0001 |
| PC(16:0/16:0) | 1050/4234 (24.8%) | 1.08 (1.03 - 1.13) | 0.001 | 1.06 (1.01 - 1.11) | 0.016 | 1.02 (0.97 - 1.08) | 0.38 |
| PC(16:0/22:5) | 1050/4234 (24.8%) | 0.87 (0.82 - 0.93) | <0.0001 | 0.86 (0.81 - 0.92) | <0.0001 | 0.87 (0.82 - 0.93) | <0.0001 |
| Cer(d18:1/24:1)/  Cer(d18:1/24:0) | 1050/4234 (24.8%) | 1.42 (1.33 - 1.50) | <0.0001 | 1.37 (1.29 - 1.45) | <0.0001 | 1.25 (1.18 - 1.33) | <0.0001 |
| Cer(d18:1/16:0)/  PC(16:0/22:5) | 1050/4234 (24.8%) | 1.26 (1.20 - 1.32) | <0.0001 | 1.27 (1.20 - 1.33) | <0.0001 | 1.25 (1.18 - 1.32) | <0.0001 |
| Cer(d18:1/18:0)/  PC(14:0/22:6) | 1050/4234 (24.8%) | 1.24 (1.17 - 1.31) | <0.0001 | 1.25 (1.18 - 1.32) | <0.0001 | 1.23 (1.17 - 1.30) | <0.0001 |
|  |  |  |  |  |  |  |  |

**^^[[2]](#footnote-2)^^**

**Table S2B.** Associations of lipid variables with CV-death, all-cause death and 3-point MACE in Cox models in GISSI-HF participants.

|  | **n_event_ /n (%)** | **Model 1** | | **Model 2** | | **Model 3** | |
| --- | --- | --- | --- | --- | --- | --- | --- |
|  |  | **HR (CI 95 %)** | **p-value** | **HR (CI 95 %)** | **p-value** | **HR (CI 95 %)** | **p-value** |
| **CV death** | | | | | | | |
| Cer(d18:1/16:0) | 242/1227 | 1.07 (0.95, 1.22) | 0.278 | 0.91 (0.75, 1.10) | 0.320 | 1.06 (0.86, 1.31) | 0.601 |
| Cer(d18:1/18:0) | 242/1227 | 0.95 (0.83, 1.07) | 0.385 | 0.92 (0.78, 1.10) | 0.383 | 0.98 (0.81, 1.19) | 0.874 |
| Cer(d18:1/24:0) | 242/1227 | 0.64 (0.57, 0.72) | <0.0001 | 0.63 (0.54, 0.73) | <0.0001 | 0.68 (0.55, 0.84) | 0.0004 |
| Cer(d18:1/24:1) | 242/1227 | 0.97 (0.85, 1.10) | 0.624 | 0.89 (0.74, 1.05) | 0.167 | 1.00 (0.83, 1.22) | 0.962 |
| PC(14:0/22:6) | 242/1227 | 0.63 (0.56, 0.71) | <0.0001 | 0.62 (0.53, 0.73) | <0.0001 | 0.67 (0.56, 0.80) | <0.0001 |
| PC(16:0/16:0) | 242/1227 | 1.12 (0.99, 1.28) | 0.070 | 0.89 (0.74, 1.08) | 0.243 | 1.00 (0.82, 1.23) | 0.975 |
| PC(16:0/22:5) | 242/1227 | 0.67 (0.59, 0.76) | <0.0001 | 0.60 (0.51, 0.72) | <0.0001 | 0.67 (0.55, 0.82) | <0.0001 |
| Cer(d18:1/24:1)/  Cer(d18:1/24:0) | 242/1227 | 1.65 (1.45, 1.88) | <0.0001 | 1.60 (1.34, 1.92) | <0.0001 | 1.40 (1.15, 1.71) | <0.0001 |
| Cer(d18:1/16:0)/  PC(16:0/22:5) | 242/1227 | 1.52 (1.35, 1.72) | <0.0001 | 1.55 (1.29, 1.87) | <0.0001 | 1.45 (1.20, 1.75) | 0.0002 |
| Cer(d18:1/18:0)/  PC(14:0/22:6) | 242/1227 | 1.44 (1.28, 1.62) | <0.0001 | 1.46 (1.25, 1.71) | <0.0001 | 1.35 (1.14, 1.59) | 0.0004 |
| **All-cause death** |  |  |  |  |  |  |  |
| Cer(d18:1/16:0) | 328/1227 | 1.11 (1.00, 1.24) | 0.055 | 1.00 (0.85, 1.17) | 0.973 | 1.16 (0.97, 1.40) | 0.099 |
| Cer(d18:1/18:0) | 328/1227 | 1.03 (0.92, 1.15) | 0.615 | 1.07 (0.92, 1.25) | 0.373 | 1.15 (0.98, 1.36) | 0.087 |
| Cer(d18:1/24:0) | 328/1227 | 0.66 (0.60, 0.73) | <0.0001 | 0.66 (0.58, 0.76) | <0.0001 | 0.75 (0.62, 0.90) | 0.002 |
| Cer(d18:1/24:1) | 328/1227 | 1.01 (0.90, 1.12) | 0.897 | 0.96 (0.83, 1.11) | 0.590 | 1.10 (0.93, 1.30) | 0.283 |
| PC(14:0/22:6) | 328/1227 | 0.65 (0.59, 0.72) | <0.0001 | 0.64 (0.56, 0.74) | <0.0001 | 0.71 (0.61, 0.83) | <0.0001 |
| PC(16:0/16:0) | 328/1227 | 1.17 (1.05, 1.30) | 0.005 | 0.97 (0.82, 1.14) | 0.677 | 1.08 (0.91, 1.29) | 0.395 |
| PC(16:0/22:5) | 328/1227 | 0.70 (0.63, 0.78) | <0.0001 | 0.65 (0.56, 0.75) | <0.0001 | 0.73 (0.62, 0.86) | 0.0002 |
| Cer(d18:1/24:1)/  Cer(d18:1/24:0) | 328/1227 | 1.50 (1.34, 1.67) | <0.0001 | 1.57 (1.36, 1.82) | <0.0001 | 1.37 (1.17, 1.61) | 0.0001 |
| Cer(d18:1/16:0)/  PC(16:0/22:5) | 328/1227 | 1.50 (1.35, 1.66) | <0.0001 | 1.55 (1.32, 1.82) | <0.0001 | 1.43 (1.22, 1.69) | <0.0001 |
| Cer(d18:1/18:0)/  PC(14:0/22:6) | 328/1227 | 1.46 (1.32, 1.61) | <0.0001 | 1.52 (1.33, 1.74) | <0.0001 | 1.39 (1.21, 1.61) | <0.0001 |
| **3-point MACE** |  |  |  |  |  |  |  |
| Cer(d18:1/16:0) | 295/1227 | 1.08 (0.96, 1.21) | 0.180 | 0.94 (0.79, 1.12) | 0.481 | 1.05 (0.86, 1.27) | 0.638 |
| Cer(d18:1/18:0) | 295/1227 | 0.98 (0.88, 1.10) | 0.786 | 0.97 (0.83, 1.14) | 0.722 | 1.01 (0.85, 1.21) | 0.871 |
| Cer(d18:1/24:0) | 295/1227 | 0.71 (0.63, 0.79) | <0.0001 | 0.70 (0.60, 0.81) | 0.013 | 0.76 (0.62, 0.92) | 0.005 |
| Cer(d18:1/24:1) | 295/1227 | 1.03 (0.92, 1.15) | 0.622 | 0.94 (0.80, 1.11) | 0.475 | 1.04 (0.87, 1.24) | 0.687 |
| PC(14:0/22:6) | 295/1227 | 0.68 (0.61, 0.76) | <0.0001 | 0.68 (0.58, 0.78) | <0.0001 | 0.71 (0.61, 0.84) | <0.0001 |
| PC(16:0/16:0) | 295/1227 | 1.09 (0.97, 1.22) | 0.148 | 0.87 (0.74, 1.04) | 0.120 | 0.93 (0.78, 1.12) | 0.464 |
| PC(16:0/22:5) | 295/1227 | 0.73 (0.65, 0.81) | <0.0001 | 0.69 (0.58, 0.80) | <0.0001 | 0.75 (0.63, 0.90) | 0.002 |
| Cer(d18:1/24:1)/  Cer(d18:1/24:0) | 295/1227 | 1.54 (1.37, 1.74) | <0.0001 | 1.47 (1.25, 1.73) | <0.0001 | 1.32 (1.10, 1.58) | 0.003 |
| Cer(d18:1/16:0)/  PC(16:0/22:5) | 295/1227 | 1.43 (1.28, 1.60) | <0.0001 | 1.47 (1.25, 1.73) | <0.0001 | 1.31 (1.10, 1.57) | 0.002 |
| Cer(d18:1/18:0)/  PC(14:0/22:6) | 295/1227 | 1.38 (1.24, 1.54) | <0.0001 | 1.38 (1.20, 1.60) | <0.0001 | 1.31 (1.13, 1.53) | <0.0001 |

^^[[3]](#footnote-3)^^

1. CI, confidence interval; CV, cardiovascular; HR, hazard ratio; MACE, major adverse cardiovascular events. Model 1: unadjusted. Model 2: adjusted for age and sex. Model 3: adjusted for age, sex, body mass index, arterial hypertension, diabetes, LVEF, eGFR and treatment group (n-3PUFA vs placebo). Model 4: model 3 + adjustment for LN-transformed NTproBNP. [↑](#footnote-ref-1)
2. Hazard ratios (HR) with 95% confidence intervals (CI) are given per 1 SD increase in log-transformed lipid variables. Model 1: unadjusted. Model 2: adjusted for age, sex. Model 3: adjusted for age, sex, body mass index, arterial hypertension, diabetes, LVEF, eGFR and treatment group (rivaroxaban vs placebo). [↑](#footnote-ref-2)
3. Hazard ratios (HR) with 95% confidence intervals (CI) is given per 1-SD increase in log-transformed lipid variables. Model 1: unadjusted. Model 2: adjusted for age and sex. Model 3: adjusted for age, sex, body mass index, arterial hypertension, diabetes, LVEF, eGFR and treatment group (n-3PUFA vs placebo). [↑](#footnote-ref-3)
